# TFE3-Mediated Autophagy is Involved in Dopaminergic Neurodegeneration in Parkinson’s Disease

**DOI:** 10.3389/fcell.2021.761773

**Published:** 2021-11-29

**Authors:** Xin He, Yue Xie, Qiongping Zheng, Zeyu Zhang, Shanshan Ma, Junyu Li, Mingtao Li, Qiaoying Huang

**Affiliations:** Guangdong Provincial Key Laboratory of Brain Function and Disease and Department of Pharmacology, Zhongshan School of Medicine, Sun Yat-sen University, Guangzhou, China

**Keywords:** Parkinson’s disease, dopaminergic neurons, autophagy, TFE3, MPTP

## Abstract

Impairment of autophagy has been strongly implicated in the progressive loss of nigral dopaminergic neurons in Parkinson’s disease (PD). Transcription factor E3 (TFE3), an MiTF/TFE family transcription factor, has been identified as a master regulator of the genes that are associated with lysosomal biogenesis and autophagy. However, whether TFE3 is involved in parkinsonian neurodegeneration remains to be determined. In this study, we found decreased TFE3 expression in the nuclei of the dopaminergic neurons of postmortem human PD brains. Next, we demonstrated that TFE3 knockdown led to autophagy dysfunction and neurodegeneration of dopaminergic neurons in mice, implying that reduction of nuclear TFE3 may contribute to autophagy dysfunction-mediated cell death in PD. Further, we showed that enhancement of autophagy by TFE3 overexpression dramatically reversed autophagy downregulation and dopaminergic neurons loss in the 1-methyl-4-phenyl-1,2,3,6-tetrahydropyridine (MPTP) model of PD. Taken together, these findings demonstrate that TFE3 plays an essential role in maintaining autophagy and the survival of dopaminergic neurons, suggesting TFE3 activation may serve as a promising strategy for PD therapy.

## Introduction

Parkinson’s disease (PD) is a multifactorial neurodegenerative disease characterized by the cardinal features of resting tremor, bradykinesia and muscle rigidity, as well as non-motor symptoms, which can develop years before motor deficits. Common pathological features of PD are selective and progressive cell death of dopaminergic neurons in the substantia nigra pars compacta (SN). However, PD is also recognized by more widespread pathology in other brain regions and it involves non-dopaminergic neurons as well. The molecular pathogenesis of PD includes various factors, such as impaired protein homeostasis, oxidative stress, mitochondria dysfunction and neuron inflammation. In recent years, growing evidence suggests that autophagy is involved in PD.

Autophagy is an essential catabolic mechanism that delivers misfolded proteins and damaged organelles to the lysosome for degradation, which is critical for maintaining neuronal survival and homeostasis (Stavoe and Holzbaur, 2019). Impaired autophagy has been verified in dopaminergic neurons of PD patients. (Karabiyik et al., 2017). Accumulation of autophagic vacuoles has been described in the SN of PD patients (Fowler and Moussa, 2018), suggesting autophagy flux disruption. Lysosomal dysfunction was indicated by decreased protein levels of Lysosomal-associated membrane protein type 1 (Lamp1), a lysosomal marker, and Cathepsin D (CatD), a lysosomal protease, in the SN of PD patients (Chu et al., 2009; Dehay et al., 2010). Lower levels of the chaperone-mediated autophagy (CMA) markers lysosomal-associated membrane protein 2A and the heat shock cognate 70 protein have also been observed in the SN of PD patients, indicating CMA dysregulation (Alvarez-Erviti et al., 2010). Moreover, genetic studies have shown that many PD-linked genes affect the degradative capacity of autophagy (Gan-Or et al., 2015). In addition, abnormal autophagy has been reported in different animal models of PD (Lu et al., 2020). For example, defects of key autophagy genes (e.g., *Atg5, Atg7, Vps35*) in mice cause dopaminergic neurodegeneration, suggesting an essential role of autophagy in the survival of dopaminergic neurons (Friedman et al., 2012; Tang et al., 2015b; Niu et al., 2016; Ren et al., 2018). More importantly, neuroprotective effects of autophagy-enhancing agents have been demonstrated in various animal models of PD (Moors et al., 2017). Thus, pharmacological activation of autophagy may be a new therapeutic strategy for PD treatment.

The microphthalmia family of bHLH-LZ transcription factors (MiT/TFE), including MITF, TFEB, TFE3 and TFEC, have been shown to positively regulate lysosomal biogenesis, lysosomal function and autophagosome formation (Steingrimsson et al., 2004; Raben and Puertollano, 2016). MiTF/TFE family members activate autophagy by binding to a specific E-box (CAXXTG) on the promoter of a variety of autophagic-lysosomal genes, including *MAP1LC3B*, *LAMP1*, *CTSD* and *SQSTM1/p62*, to promote their expression (Settembre et al., 2011; Martina et al., 2014; Moller et al., 2019). TFEB, a member of the MiTF/TFE family, has received considerable attention and its dysfunction has been implicated in the pathogenesis of several neurodegenerative diseases (Cortes and La Spada, 2019). The protective effects of autophagy enhancement by TFEB activation have been verified in PD animal models. (Decressac et al., 2013; Torra et al., 2018; Zhuang et al., 2020). However, whether and how other members of MiTF/TFE family are involved in PD pathogenesis remains unknown. One study showed that *TFE3* mRNA levels were the highest in the MiTF/TFE family in human brain tissue, about three times higher than TFEB (Kuiper et al., 2004). Another study reported that *TFE3* mRNA levels are higher than those of *TFEB* in human hippocampus tissue, and it further demonstrated that TFE3 but not TFEB plays a prominent role in promoting lysosomal biogenesis and autophagy upregulation in CA1 neurons during Alzheimer’s disease pathogenesis (Bordi et al., 2016). These studies imply that TFE3, with a very high abundance of mRNA, may play a pivotal role in the central nervous system. Therefore, this study aimed to investigate the role of TFE3 in autophagy of dopaminergic neurons and in PD pathogenesis.

## Materials and Methods

### Human Brain Samples

Human tissue was obtained from the University of Miami Brain Endowment Bank (NIH, Bethesda, MD, United States). Brain sections were paraffin embedded and supplied at 5-μm thickness. For immunohistochemistry, we studied samples from five PD patients and five age-matched controls (Table 1). The average age and postmortem intervals for PD patients and controls were not significantly different (two-tailed Student’s *t* test. Age: *p* = 0.3713. Postmortem intervals: *p* = 0.2012).

**TABLE 1:** Parkinson’s disease patients and control subjects data.

Tissue Code	Type of Fixed Biospecimens	Brain Type	Sex	Age	Race	Autolysis (hr)
991_HBGQ_01_18_SN	Mid-Brain Substantia Nigra	Parkinson’s Disease	F	90	White	23.24
991_HBCX_01_18_SN	Mid-Brain Substantia Nigra	Parkinson’s Disease	F	81	White	32.40
991_HBMD_01_18_SN	Mid-Brain Substantia Nigra	Parkinson’s Disease	F	79	White	25.00
991_HBHB_01_18_SN	Mid-Brain Substantia Nigra	Parkinson’s Disease	M	77	White	24.00
991_HBDK_01_18_SN	Mid-Brain Substantia Nigra	Parkinson’s Disease	M	79	White	38.60
991_HctZA_01_18_SN	Mid-Brain Substantia Nigra	Unaffected Control	F	79	White	17.80
991_HctZZC_01_18_SN	Mid-Brain Substantia Nigra	Unaffected Control	F	82	White	14.20
991_Hct15HAC_01_18_SN	Mid-Brain Substantia Nigra	Unaffected Control	F	90	White	31.50
991_HctZZV_01_18_SN	Mid-Brain Substantia Nigra	Unaffected Control	F	86	White	25.11
991_Hct15HBC_01_18_SN	Mid-Brain Substantia Nigra	Unaffected Control	M	83	White	25.00

### Animals

Eight-to 12-week-old male C57BL/6 mice weighing 22–28 g were purchased from the Beijing Vital River Laboratory Animal Technological Company (Beijing, China). All mice were housed in specific pathogen-free facilities, maintained on a 12-h light/dark cycle with controlled temperature and air humidity, and allowed free access to food and water. All experimental procedures were approved by the Institutional Animal Care and Use Committee (IACUC), Sun Yat-Sen University, Guangzhou, China. Mice were assigned randomly for treatment.

### Adeno-Associated Virus Production and Infection

rAAV2/9-TH-Tfe3-3×Flag-WPRE (AAV-TFE3) contained the murine *Tfe3* cDNA fused to that of the 3×Flag epitope, and rAAV2/9-TH-3×Flag-WPRE (AAV-Flag) containing only the cDNA of 3×Flag epitope was used as a control. rAAV2/9-hSyn-EGFP-miR30a-shTfe3-WPRE (AAV-shTFE3) was used to target murine *Tfe3* gene and rAAV2/9-hSyn-EGFP-miR30a-shScramble-WPREs (AAV-shScr) was used as a control. The *Tfe3* shRNA sequence was 5′-GCG​ACA​GAA​GAA​AGA​CAA​TCA-3’. The scrambled shRNA sequence was 5′-CCT​AAG​GTT​AAG​TCG​CCC​TCG-3′, non-target in mice. All the viruses were generated and packaged by BrainVTA (Wuhan, China) and PackGene Biotech (Guangzhou, China). The titer of all the viruses in this study was 1x10^12^ gc/mL. The AAV viruses were delivered at 100 nl/min unilaterally into the right SN. The coordinates indicating distance (mm) from the bregma were as follows: anteroposterior −2.9, mediolateral +1.3, and dorsoventral −4.35. After the injection, the needle remained in place for at least 5 min to prevent retrograde flow along the needle track. After surgery, the mice were warmed under a heating pad until they awakened.

### Drug Administration

MPTP (Sigma, Shanghai, China) treatments were performed as previously described (Li et al., 2020). Briefly, ten-to 12-week-old male mice were intraperitoneally injected (i.p.) with MPTP (dissolved in saline, 30 mg/kg body weight) or saline for 5 days at 24 h intervals. The mice were intraperitoneally injected with MPTP 1 week after the stereotaxic administration of the AAV virus. For immunofluorescence and immunohistochemistry, the mice were sacrificed 1 day and 21 days after the last MPTP injection, respectively.

### Tissue Preparation

For real-time PCR, mice were sacrificed by cervical dislocation and the brains were rapidly removed and washed with ice-cold PBS. The SN tissue was rapidly dissected on ice and stored at −80°C prior to further experiments. For immunofluorescence and immunohistochemistry, mice were anesthetized with chloral hydrate (400 mg/kg, i. p.) and then intracardially perfused with ice-cold PBS for 3 min, followed by cold 4% PFA at a flow rate of 10 ml/min for 8 min. Mouse brains were removed and post-fixed overnight in 4% PFA at 4°C and then immersed in 20% and 30% sucrose. Tissues were embedded in embedding media O.C.T (#4583, SAKURA) and cut into 20 μm-thick cryosections for immunofluorescence and 40 μm-thick cryosections for immunohistochemistry.

### Real-Time PCR

Total RNA was extracted from the SN tissue using TRIzol reagent (Thermo Fisher, United States), and the RNA concentration was determined spectrophotometrically (Beckman DU 730). NovoScript^®^ Plus All-in-one 1st Strand cDNA Synthesis SuperMix (gDNA Purge) (E047-01B, novoprotein, China) was used to synthesize the cDNA, which was then amplified using a NovoStart^®^ SYBR qPCR SuperMix Plus (E096-01A, novoprotein, China) with specific primers for real-time PCR analysis. All reactions were carried out using the Light Cycler 480 System (Roche, Switzerland). The following primer sequences were used: *Tfe3*, forward: 5′- ATC​TCT​GTG​ATT​GGC​GTG​TCT-3′, reverse: 5′-GAA​CCT​TGA​GTA​CCT​CCC​TGG- 3'; *Map1lc3b*, forward: 5′- CGC​TTG​CAG​CTC​AAT​GCT​AAC-3′, reverse: 5′- CTC​GTA​CAC​TTC​GGA​GAT​GGG-3′; *Lamp1*, forward: 5′-TGC​TCC​GGG​ATG​CCA​CTA​T-3′, reverse: 5′- TGT​TGT​CCT​TTT​TCA​GGT​AGG​TG-3′; *Ctsd*, forward: 5′- CGA​TTA​TCA​GAA​TCC​CTC​TGC​G-3′, reverse: 5′- GGT​CTT​AGG​CGA​TGA​CTG​CAT-3′; *Sqstm1/p62*, forward: 5′- GAA​CTC​GCT​ATA​AGT​GCA​GTG​T-3′, reverse: 5′- AGA​GAA​GCT​ATC​AGA​GAG​GTG​G-3'; *Actin*, forward: 5′-GGC​TGT​ATT​CCC​CTC​CA TCG-3′, reverse: 5′-CCA​GTT​GGT​AAC​AAT​GCC​ATG​T-3'.

### Immunofluorescence

Immunofluorescence analysis was performed as previously described (Li et al., 2020). Briefly, free-floating sections were pre-incubated in blocking solution containing 5% normal donkey serum at room temperature for 1 h. Then primary antibodies were dissolved in diluent and incubated with sections overnight at 4°C. The following primary antibodies were used: TH (1:1,000, #AB9702, Millipore), TFE3 (1:500, #ab93808, Abcam), LC3 (1:100, #2775, Cell signaling Technology), Lamp1 (1:1,000, #1D4B-C, DSHB), CatD (1:200, #SC-6486, Santa Cruz Biotechnology), and p62 (1:1,000, #GP62-C, Progen Biotechnik). After three washes, the sections were then incubated with the secondary antibodies (Thermo Fisher), which were conjugated with Alexa 488, Alexa 555, or Alexa 647, at room temperature for 1 h. Finally, the sections were visualized under a confocal laser scanning microscope (LSM 780, Carl Zeiss) and the immunofluorescent images were quantified using ImageJ software.

### Immunohistochemistry

For human samples, paraffin-embedded human brain tissue slices were deparaffinized in xylene and rehydrated in graded alcohols. Antigens were retrieved by microwave heating with citrate buffer (pH 6) for a total of 30 min. Then, tissue sections were incubated in 3% H_2_O_2_ at room temperature for 30 min to block nonspecific peroxidase-activity and then blocked in 2.5% normal horse blocking serum for 1 h at room temperature. Tissue sections were first incubated with Iba1 (1:1,000, #ab5076, Abcam) overnight at 4°C, washed, incubated with the secondary antibody (#MP-5405, Vector Labs) for 1 h at room temperature, and then visualized using an ImmPACT Vector Red substrate kit (#SK-5105, Vector Labs). Subsequently, the tissue sections were similarly incubated with TFE3 (1:500, #HPA023881, Sigma) and the secondary antibody (#MP-7401, Vector Labs) and visualized by DAB (#SK-4100, Vector Labs). Next, tissue sections were dehydrated in graded alcohols and xylene before mounting with permanent mounting medium (#H-5000, Vector Labs). Finally, the sections were examined under a light microscope (Eplice Ni-E, Nikon).

The optical density of TFE3 staining on human brain samples was analyzed with the aid of Image Pro Plus 6 software. The regions of the nuclei and cytoplasm of neuromelanin-positive cells were defined by experienced researchers. The area with neuromelanin was excluded to avoid the interference of neuromelanin. The density of DAB staining in the nuclear and cytoplasmic regions was measured separately, and then the ratio of nuclear/cytoplasmic TFE3 expression was calculated in each cell.

For mouse samples, the immunohistochemical method was performed as previously described (Li et al., 2020). Briefly, cryo-coronal sections (40 μm) encompassing the entire midbrain and striatum were collected serially. Sections were incubated with anti-TH antibody (1:10,000, #AB152, Merck Millipore) overnight at 4°C and then visualized using the Vectastain Elite ABC Kit (#PK-6101, Vector Labs) and the DAB Peroxidase Substrate Kit (#SK-4100, Vector Labs), following the manufacturer’s protocol.

For Nissl staining, adjacent midbrain sections were mounted on the slides, stained with 0.5% cresyl violet after de-fatting, dehydrated and cover-slipped.

### Stereological Cell Counting and Densitometric Analysis

To estimate the number of TH-positive and Nissl-positive neurons in the SN, stereological counting was performed as previously described (Li et al., 2020). Briefly, stereological cell counting analyses were performed using a stereological method of optical fractionator with the aid of Stereo Investigator (MicroBrightField Inc.). Every fourth 40 μm-thick sections from the midbrain (AP −2.7 to −3.8 mm) were collected. The SN was delineated under a ×4 objective, and the actual counting was performed under a ×100 objective. Stereological counting was performed in a double-blind fashion. The optical density of striatal TH-positive fibers was estimated using ImageJ software. The region of the dorsal striatum was determined as previously described (Li et al., 2020). All analyses were performed blinded to the treatments.

### Statistical Analysis

GraphPad Prism version 8.0 (GraphPad Software) was used for the statistical analysis. All data are presented as mean ± standard error of the mean (SEM). When comparing data from two groups, a two-tailed Student’s *t* test was used. When there were two variables, ANOVA followed by Tukey’s multiple comparisons test was used. For all analyses, statistical significance was considered when probability value of *p* < 0.05.

## Results

### Nuclear TFE3 Expression Is Reduced in the SN Dopaminergic Neurons of Parkinson’s Disease Patients

**FIGURE 1:**
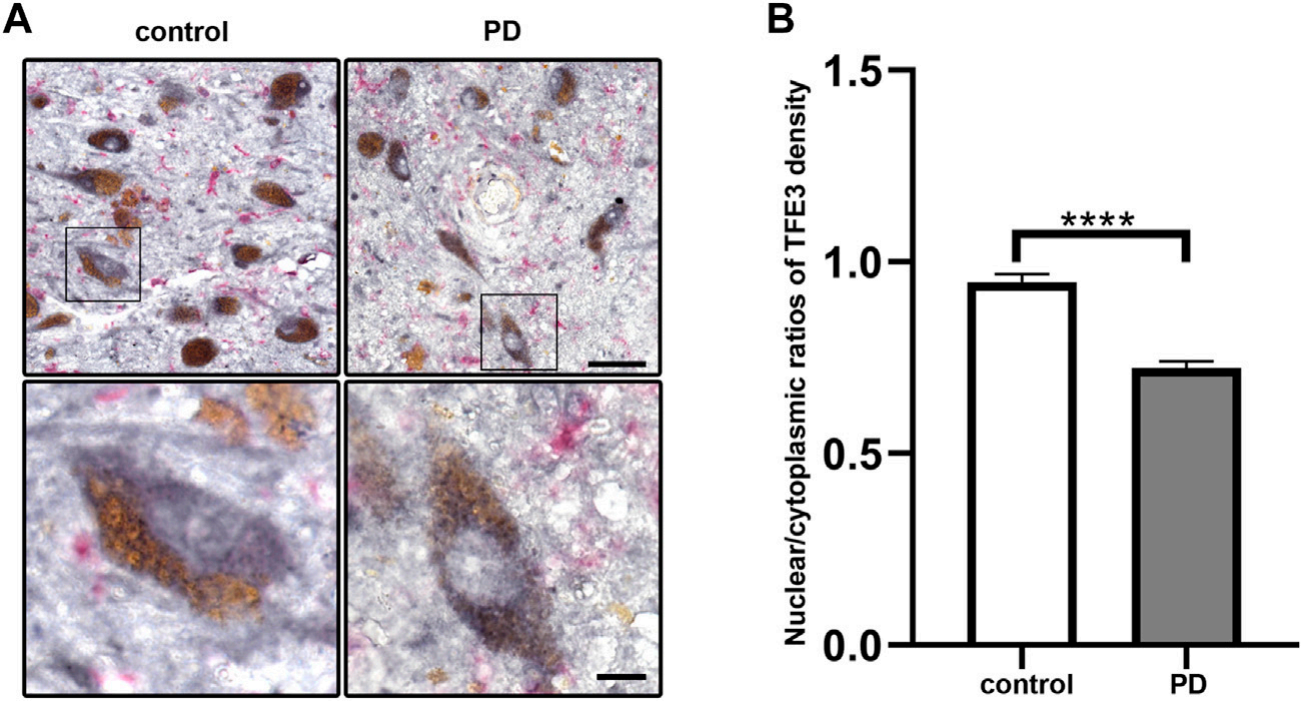
Nuclear TFE3 expression is reduced in the SN dopaminergic neurons of PD patients. **(A)** Sections of human SN obtained from PD or control subjects were immunostained using a TFE3 antibody visualized by DAB staining (gray-black) and a Iba1 antibody visualized by ImmPACT Vector Red staining (magenta). Scale bars: upper panel: 50 μm; lower panel: 10 μm. **(B)** Quantification of the nuclear to cytoplasmic ratios of TFE3 in human SN dopaminergic neurons. n = 436 dopaminergic neurons from 5 PD patients and 898 dopaminergic neurons from five controls, respectively. Data are expressed as the mean ± SEM. ****p < 0.0001, two-tailed Student’s *t* test.

To investigate whether TFE3 is involved in the pathogenesis of PD, we first analyzed TFE3 protein expression levels in postmortem human brains. Immunohistochemistry for TFE3 was performed on the midbrain sections of postmortem brains from both PD and control subjects. The brownish pigment neuromelanin served as a convenient marker for dopaminergic neurons, and TFE3 immunostaining was visualized with gray-black substrate (DAB with Nickel) to best distinguish it from neuromelanin. TFE3 staining was evident in the SN dopaminergic neurons of control brains and it was localized to the nucleus and cytoplasm (Figure 1A). In PD patients, we found that TFE3 staining in the nuclei was reduced in the SN dopaminergic neurons (Figure 1A).

The shuttling of MiTF/TFE family members between the nucleus and cytoplasm usually occurred in a variety of stress condition (Raben and Puertollano, 2016). Therefore, we performed quantitative analysis of nuclear-to-cytoplasmic TFE3 immunostaining in individual neuromelanin-positive neurons in five PD and five control subjects. The quantitative results showed that the nuclear/cytoplasmic ratios of TFE3 were decreased in PD patients, demonstrating a reduction of nuclear TFE3 expression in the dopaminergic neurons of PD patients (Figure 1B). These results suggest that the transcriptional activity of TFE3 is repressed in SN dopaminergic neurons of PD patients.

### TFE3 Knockdown Leads to Autophagy Flux Disruption in Dopaminergic Neurons

Although TFE3 is reported to be a crucial gene that regulates autophagy and lysosomal biogenesis, the function of TFE3 in dopaminergic neurons is undefined. Therefore, we assessed whether TFE3 is involved in autophagy of dopaminergic neurons by TFE3 knockdown. We delivered AAV-shScr or AAV-shTFE3 to the unilateral SN of mice by stereotaxic injections. Five weeks following AAV injection, the mice were sacrificed.

**FIGURE 2:**
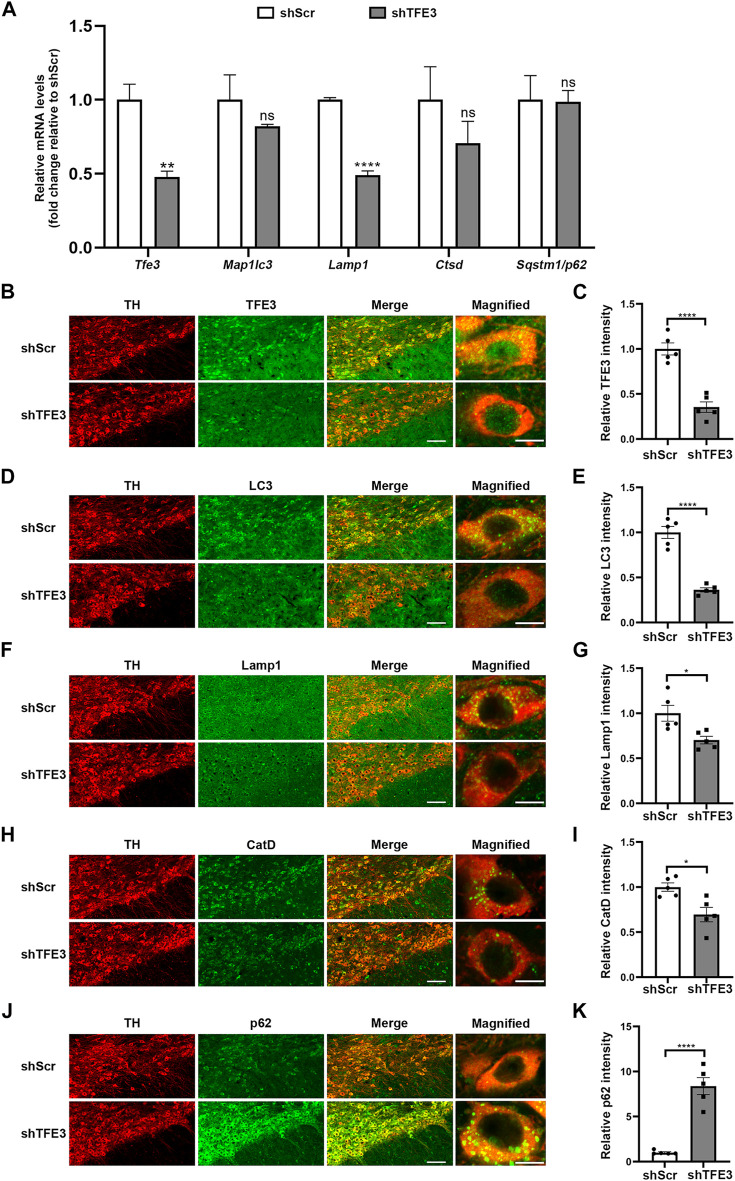
TFE3 knockdown impairs autophagy of dopaminergic neurons. **(A)** Quantitative RT-PCR analysis of *Tfe3*, *Map1lc3*, *Lamp1*, *Ctsd* and *Sqstm1/p62* mRNA in the SN of mice injected with AAV-shScr or AAV-shTFE3. n = 3 per group. **(B, D, F, H and J)** Representative images showing immunofluorescence for TFE3 (green, B), LC3 (green, D), Lamp1 (green, F), CatD (green, H) and p62 (green, J) in SN dopaminergic neurons (TH-positive, red) of mice injected with AAV-shScr or AAV-shTFE3. n = 5 per group. Scale bars: low magnification, 100 μm; high magnification, 10 μm. **(C, E, G, I and K)** Quantitative data are shown. Data are expressed as the mean ± SEM. *p < 0.05, **p < 0.01, ****p < 0.0001, ns, not significant, two-tailed Student’s *t* test.

AAV-shTFE3 injection significantly decreased *Tfe3* mRNA levels in the SN of mice (Figure 2A). Then, we measured the transcriptional levels of autophagy-lysosomal genes targeted by TFE3. The mRNA levels of *Map1lc3b*, *Lamp1* and *Cstd* were diminished in the AAV-shTFE3 group, although only the decrease in *Lamp1* was statistically significant (Figure 2A). No significant change was observed in the mRNA levels of *Sqstm1/p62* (Figure 2A). To precisely assess the effect of TFE3 knockdown on autophagy of dopaminergic neurons, we observed the autophagy-lysosomal proteins *in situ* by immunofluorescence. Our results showed that TFE3 was distributed in both the cytoplasm and the nuclei of dopaminergic neurons (Figure 2B), as observed in human (Figure 1A). TFE3 of dopaminergic neurons was dramatically reduced in the AAV-shTFE3 group (Figures 2B,C). LC3, an autophagosome marker, was significantly decreased in the dopaminergic neurons of mice injected with AAV-shTFE3 compared with AAV-shScr (Figures 2D,E). The magnified image showed that the number of LC3-positive dots of dopaminergic neurons was also markedly reduced in the AAV-shTFE3 group (Figure 2D), implicating a blockade of autophagosome formation by TFE3 knockdown . Lamp1, a lysosome marker, and CatD, a lysosomal protease, showed decreased expression levels in dopaminergic neurons of mice injected with AAV-shTFE3 (Figure 2F–I). Similarly, magnified images showed that the numbers of Lamp1-and CatD-positive dots of dopaminergic neurons were reduced in the AAV-shTFE3 group (Figures 2F,H), indicating that the number and activity of lysosomes were reduced by TFE3 knockdown. Conversely, p62, an autophagy receptor, was significantly up-regulated in the dopaminergic neurons of mice injected with AAV-shTFE3 (Figures 2J,K). Furthermore, magnified image showed that TFE3 knockdown promoted p62 aggregates formation (Figure 2J), suggesting compromised autophagic degradative capacity. These results demonstrate that TFE3 inhibition of dopaminergic neurons leads to autophagy flux disruption, supporting the notion that reduced nuclear TFE3 in dopaminergic neurons may result in the downregulation of autophagy in PD patients.

### TFE3 Knockdown Induces Degeneration of Dopaminergic Neurons

**FIGURE 3:**
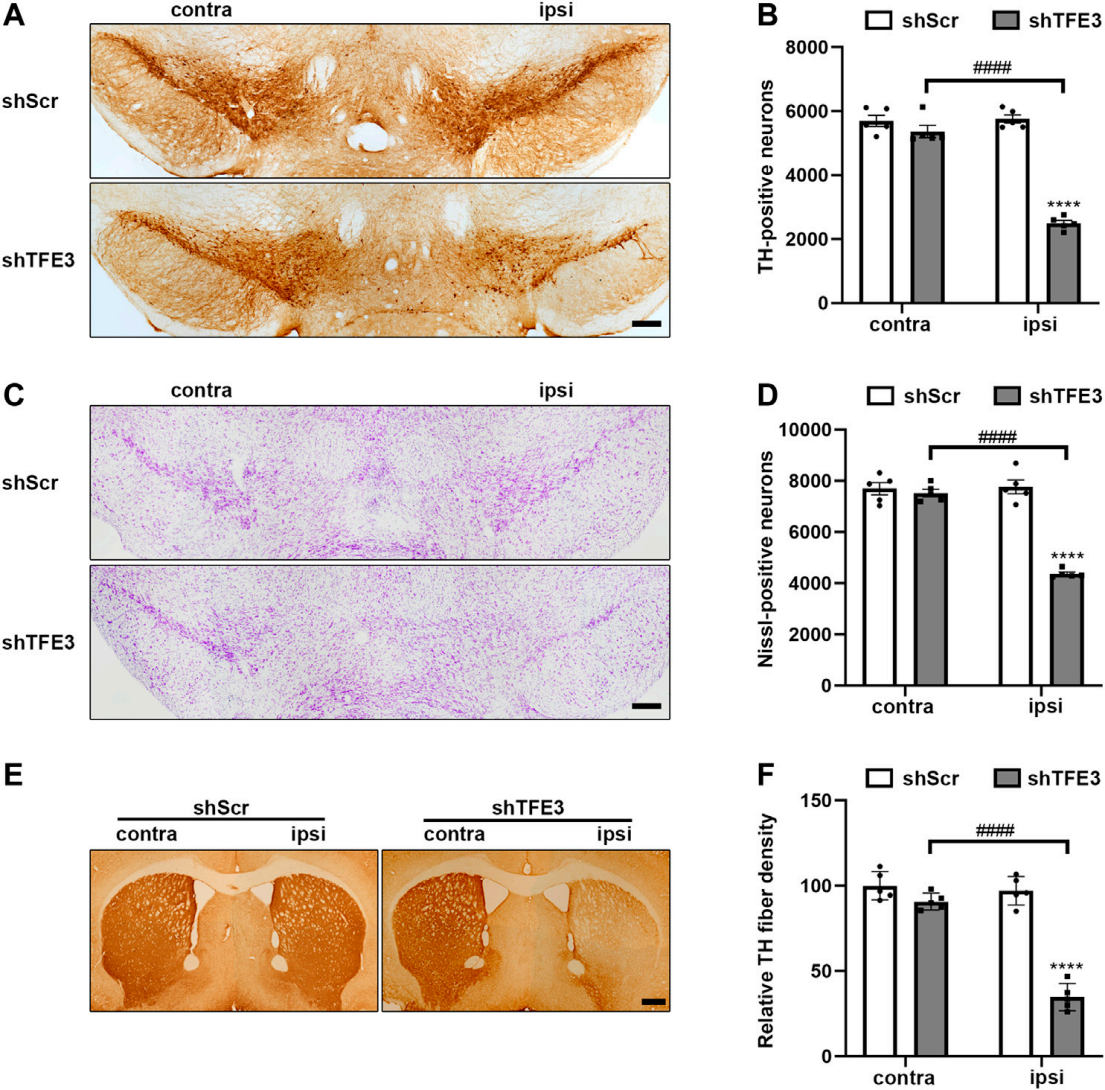
TFE3 knockdown induces degeneration of dopaminergic neurons. **(A, C)** Immunohistochemical TH staining **(A)** and Nissl staining **(C)** of midbrain sections in mice after AAV-shScr or AAV-shTFE3 injection. Scale bars: A, C 200 μm. **(B, D)** Stereological cell counts of TH-positive **(B)** and Nissl-positive **(D)** neurons in the SN. **(E)** Immunohistochemical TH staining of striatal sections in mice after AAV-shScr or AAV-shTFE3 injection. Scale bars: 500 μm. **(F)** Quantitation of optical densities of the striatal TH immunostaining; n = 5 per group. Data are expressed as the mean ± SEM. ****p < 0.0001, ####*p* < 0.0001, ANOVA followed by Tukey’s multiple comparisons test.

Previous studies show that defective autophagy often causes dopaminergic neurons loss (Friedman et al., 2012; Tang et al., 2015a; Niu et al., 2016; Ren et al., 2018). Since autophagy was severely impaired in dopaminergic neurons by TFE3 knockdown, we examined the impact of TFE3 knockdown on dopaminergic neuronal survival. We performed an unbiased stereological analysis of dopaminergic neurons in the SN of mice 3 months after the stereotaxic administration of AAV-shScr or AAV-shTFE3. Unbiased stereological estimation revealed a 56.7% loss of TH-positive neurons in the AAV-shTFE3 group relative to the AAV-shScr group (Figures 3A,B). Consistently, there was a 43.8% reduction of Nissl-positive neurons in the SN in the AAV-shTFE3 group compared with the AAV-shScr group (Figures 3C,D), suggesting an actual dopaminergic neuronal loss rather than a loss of TH expression. In parallel with the results in the SN, densitometric analysis showed that TFE3 knockdown resulted in a 64.3% decrease in TH-positive fibers in the striatum (Figures 3E,F). Taken together, these results indicate that TFE3-mediated autophagy is required for the survival of dopaminergic neurons.

### TFE3 Overexpression Enhances Autophagy of Dopaminergic Neurons

Because TFE3 knockdown contributes to autophagy dysfunction of dopaminergic neurons, we investigated whether TFE3 overexpression can enhance autophagy in dopaminergic neurons. To activate TFE3, we delivered Flag-tagged murine *Tfe3* under the TH promoter by AAV (AAV-TFE3). AAV-Flag was used as a control. Both viruses were injected unilaterally in the SN of mice. Two weeks after AAV injection, the mice were sacrificed.

**FIGURE 4:**
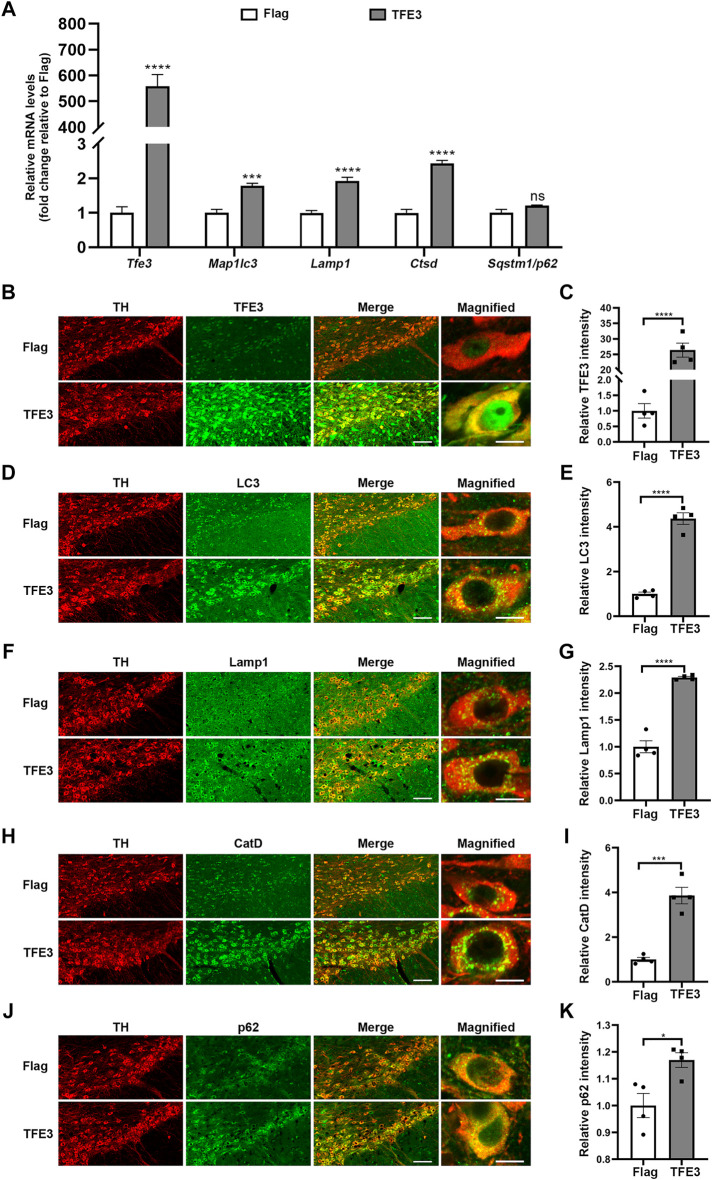
TFE3 overexpression enhances autophagy in dopaminergic neurons. **(A)** Quantitative RT-PCR analysis of *Tfe3*, *Map1lc3*, *Lamp1*, *Ctsd* and *Sqstm1/p62* mRNA in the SN of mice injected with AAV-Flag or AAV-TFE3. n = 5 per group. **(B, D, F, H and J)** Representative images showing immunofluorescence for TFE3 (green, B), LC3 (green, D), Lamp1 (green, F), CatD (green, H) and p62 (green, J) in SN dopaminergic neurons (TH-positive, red) of mice injected with AAV-Flag or AAV-TFE3. n = 4 per group. Scale bars: low magnification, 100 μm; high magnification, 10 μm. **(C, E, G, I and K)** Quantitative data are shown. Data are expressed as the mean ± SEM. *p < 0.05, ***p < 0.001, ****p < 0.0001, ns, not significant, two-tailed Student’s *t* test.

Contrary to TFE3 knockdown, AAV-TFE3 injection significantly increased *Tfe3* mRNA levels in the SN of mice (Figure 4A). Autophagy-lysosomal genes reported to be targeted by TFE3 were up-regulated, except the increase in *Sqstm1/p62* was not statistically significant (Figure 4A). Likewise, to precisely examine the effect of TFE3 overexpression on autophagy of dopaminergic neurons, we observed the autophagy-lysosomal proteins *in situ* by immunofluorescence. We found that TFE3 protein expression was remarkably upregulated in the dopaminergic neurons of mice injected with AAV-TFE3 compared with AAV-Flag (Figures 4B,C). In contrast to TFE3 knockdown, the protein expression levels of LC3, Lamp1 and CatD were significantly increased in the dopaminergic neurons of mice injected with AAV-TFE3 (Figure 4D–I). Moreover, magnified images showed that the numbers of LC3-, Lamp1-and CatD-positive dots were increased, demonstrating that TFE3 overexpression promoted autophagosome formation and lysosomal biogenesis (Figures 4D,F,H). Although a slight increase of p62 was also observed in the AAV-TFE3 group, magnified images showed that there were no p62 aggregates (Figures 4J,K). These findings indicate that TFE3 overexpression enhances autophagy flux in dopaminergic neurons.

### TFE3 Overexpression Reverses the Downregulation of Autophagy in the MPTP Model

Previous studies showed lysosomal depletion and reduced autophagosomes in the MPTP mouse model of PD (Dehay et al., 2010; Bove et al., 2014; Zhu et al., 2020). Our results showed that TFE3 overexpression can activate the biogenesis of autophagosomes and lysosomes. Hence, an MPTP mouse model was used to determine whether TFE3 overexpression could counteract autophagy defects in dopaminergic neurons. One week after AAV injection, the mice were challenged with MPTP. For immunofluorescence analysis, mice were sacrificed 1 day after the last MPTP injection.

**FIGURE 5:**
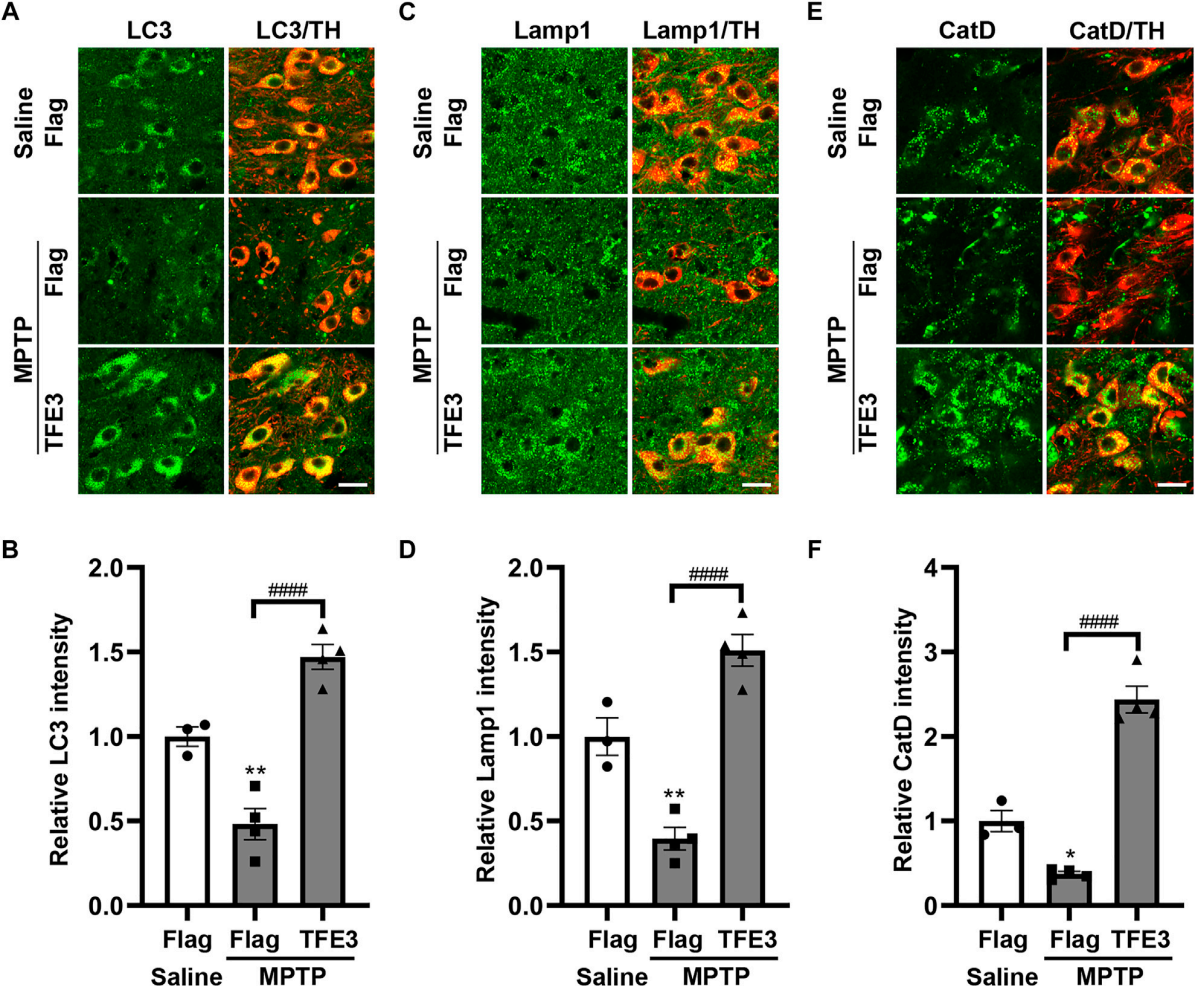
TFE3 overexpression rescues the autophagy defects of dopaminergic neurons in MPTP-treated mice. **(A, C and E)** Detection of LC3 (green, A), Lamp1 (green, C) and CatD (green, E) in dopaminergic neurons (red) of the SN from Saline- and MPTP-injected mice overexpressing Flag or TFE3. Scale bars: 20 μm. **(B, D and F)** Quantitative data are shown. n = 3-4 per group. Data are expressed as the mean ± SEM. *p < 0.05, **p < 0.01, ####*p* < 0.0001, ANOVA followed by Tukey’s multiple comparisons test.

In mice injected with AAV-Flag, MPTP resulted in decreased protein expression levels of LC3 compared with those in Saline-treated mice (Figures 5A,B). TFE3 overexpression completely reversed MPTP-induced downregulation of LC3 in dopaminergic neurons (Figures 5A,B). Similarly, TFE3 overexpression significantly rescued the decrease of Lamp1 and CatD in the dopaminergic neurons of MPTP-treated mice, compared to the AAV-Flag group (Figures 5C–F). These results demonstrate that TFE3 overexpression can reverse MPTP-induced autophagy dysfunction in dopaminergic neurons.

### TFE3 Overexpression Alleviates MPTP-Induced Dopaminergic Neurodegeneration

Our results showed that TFE3 overexpression restored autophagic activity of dopaminergic neurons in the MPTP model. Activation of autophagy in dopaminergic neurons often exerts a neuroprotective effect. Therefore, we evaluated whether TFE3 overexpression has a neuroprotective effect. For immunohistochemical analysis, mice were sacrificed 21 days after the last MPTP injection.

**FIGURE 6:**
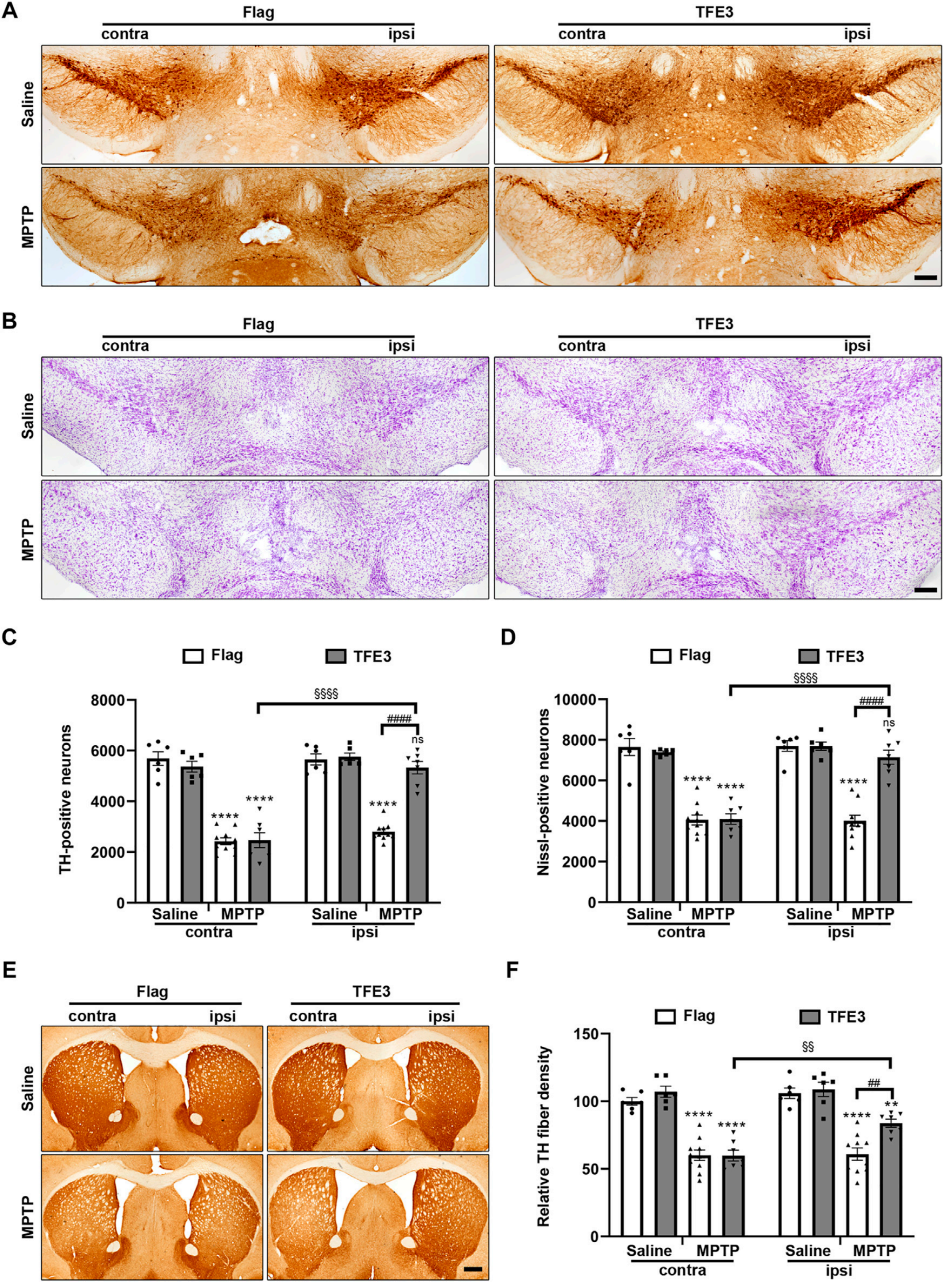
TFE3 overexpression alleviates dopaminergic neurons loss from MPTP neurotoxicity. **(A, B)** Immunohistochemical TH staining (A) and Nissl staining (B) of midbrain sections from Saline- and MPTP-treated mice overexpressing Flag or TFE3. Scale bars: A, B 200 μm. **(C, D)** Stereological cell counts of TH-positive (C) and Nissl-positive (D) neurons in the SN. **(E)** Immunohistochemical TH staining of striatal sections from Saline- and MPTP-treated mice overexpressing Flag or TFE3. Scale bars: 500 μm. **(F)** Quantitation of optical densities of the striatal TH immunostaining; n = 6–10 per group. Data are expressed as the mean ± SEM. **p < 0.01, ****p < 0.0001, ##*p* < 0.01, ####*p* < 0.0001, §§*p* < 0.01, §§§§*p* < 0.0001, ns, not significant, ANOVA followed by Tukey’s multiple comparisons test.

We systematically analyzed dopaminergic neuron numbers in the SN of mice after unilateral injection of AAV viruses. Unbiased stereological estimation revealed a 50.5% loss of TH-positive neurons induced by MPTP injection in the AAV-Flag group, while only 7.4% of the TH-positive neurons degenerated in the AAV-TFE3 group (Figures 6A,C). Nissl staining revealed similar trends and statistical results (47.8 and 7.1% loss in the AAV-Flag and AAV-TFE3 groups, respectively; Figures 6B,D), suggesting an actual dopaminergic neuronal loss rather than a loss of TH expression. The loss of dopaminergic neurons in the SN was accompanied by fewer TH-positive terminals in the striatum. Next, we quantified TH-positive terminals in the striatum using densitometry. In parallel with the results in the SN, TFE3 overexpression mitigated MPTP-induced loss of TH-positive fibers in the striatum (Figures 6E,F). These findings demonstrate that TFE3 overexpression prevents MPTP-induced dopaminergic neurodegeneration.

## Discussion

In this study, we provided the first evidence that nuclear TFE3 expression was decreased in nigral dopaminergic neurons of PD patients. We then demonstrated, for the first time, that TFE3 could regulate autophagy in dopaminergic neurons by knockdown and overexpression of TFE3. Further, our results showed that autophagy dysfunction by TFE3 knockdown led to dopaminergic neurons loss, while autophagy enhancement by TFE3 overexpression rescued MPTP-induced autophagy defects and prevented the dopaminergic neurons death.

Autophagy is essential for maintaining dopaminergic neurons survival by removing abnormal aggregated proteins and dysfunctional organelles, including α-synuclein aggregates and damaged mitochondria, which can produce parkinsonian features (Mullin and Schapira, 2013). However, the mechanism underlying autophagy defects in PD has not been fully elucidated. We showed that TFE3 expression in the nucleus was reduced in the dopaminergic neurons of patients with PD. Moreover, our results showed that TFE3 knockdown led to autophagy dysfunction by downregulating the protein expression levels of Lamp1 and CatD and increasing p62 aggregations in dopaminergic neurons, which were also shown in postmortem brains of patients with PD (Kuusisto et al., 2003; Chu et al., 2009). These results suggest that reduced nuclear TFE3 in dopaminergic neurons may contribute to autophagic impairment in PD. Autophagy is often critical for the survival of dopaminergic neurons. A deficiency in the key autophagy gene leads to dopaminergic neurons loss (Friedman et al., 2012; Tang et al., 2015a; Niu et al., 2016; Ren et al., 2018). We further demonstrated that TFE3 knockdown significantly caused the degeneration of dopaminergic neurons, implying that reduction of TFE3 in nuclei could contribute to autophagy dysfunction-mediated dopaminergic neuron death in PD.

The RT-PCR and immunofluorescence results showed some discrepancies. Although shTFE3 significantly decreased *Tfe3* mRNA levels by RT-PCR, the decrease in *Map1lc3b* and *Cstd* mRNA levels was not significant, while a clear decrease of LC3 and CatD in dopaminergic neuron was revealed by immunofluorescence. The shTFE3 was driven by a neuron-specific promoter, and thus only infected neurons but not glial cells could express the shRNA (data not shown). The genes examined in RT-PCR were universally expressed in the glial cells surrounding dopaminergic neurons in the SN, which were inevitably collected during tissue dissection. Therefore, it is possible that the *Map1lc3b* and *Cstd* mRNA levels of glia in the SN caused the discrepancy between the RT-PCR and immunofluorescence results. The mRNA levels of *Sqstm1/p62* were not significantly changed by either TFE3 knockdown or TFE3 overexpression, suggesting *Sqstm1/p62* may not be a target gene of TFE3 in dopaminergic neurons. Immunofluorescent staining showed p62 protein levels were increased by TFE3 knockdown, with obvious p62 aggregation in the dopaminergic neurons, indicating reduced autophagic degradative capacity induced by TFE3 knockdown. TFE3 overexpression, which enhanced autophagic activity and should have led to a decline in p62 expression, also up-regulated p62 protein levels in the dopaminergic neurons. This may be due to a strong increase of MiTF/TFE functions caused by TFE3 overexpression.

TFE3 and TFEB, another MiTF/TFE family member, are partially redundant in terms of their ability to induce lysosomal biogenesis and autophagy (Raben and Puertollano, 2016). Our results showed that knockdown of TFE3 alone led to autophagy dysfunction and the death of dopaminergic neurons, suggesting a predominant role of TFE3. Some studies have shown that *TFE3* mRNA levels are the highest in the MiTF/TFE family in human brain tissue (Kuiper et al., 2004), and the expression of *TFE3* is ten times higher than *TFEB* in human hippocampus tissue (Bordi et al., 2016), suggesting that TFE3 may be the dominant one among MiTF/TFE family in neurons. Nuclear localization is a prerequisite for transcriptional activity of a transcription factor. Our results showed that TFE3 was distributed in both the nuclei and cytoplasm of dopaminergic neurons in humans and mice, while TFEB was mainly distributed in the cytoplasm of dopaminergic neurons in mice (data not shown), similar to other reported results (Siddiqui et al., 2015; Zhuang et al., 2020). Based on the basal expression level and subcellular localization, it is possible that TFE3 plays a major role in maintaining autophagy and survival in dopaminergic neurons.

It has been reported that autophagy activity was impaired in the MPTP model (reviewed in (Lu et al., 2020)). We found that TFE3 overexpression increased the protein levels of LC3, Lamp1, CatD and p62, suggesting that TFE3 activation can enhance autophagy flux. Further, our results showed that TFE3 overexpression reversed MPTP-induced autophagy dysfunction. Autophagy activation has been shown to be neuroprotective in preclinical models of PD (reviewed in (Fowler and Moussa, 2018)). As expected, we found that TFE3 overexpression protected dopaminergic neurons against MPTP neurotoxicity, demonstrating that TFE3 activation can confer neuroprotection in PD animal model.

Although TFE3 plays a neuroprotective role in the MPTP model, the detailed mechanism remains unclarified. Previous studies have shown that impairment of mitochondria and lysosome may be the underlying mechanism of neuronal cell death in PD (Dehay et al., 2010; Bose and Beal, 2016). TFE3 overexpression may enhance autophagy to remove damaged mitochondria and lysosomes, inhibiting mitochondrial-dependent apoptosis and lysosomal membrane permeabilization (LMP)-mediated cell death in dopaminergic neurons (Bove et al., 2014; Langston, 2017). In addition, a recent report indicated that TFE3 overexpression augments autophagy flux and ameliorates ER stress-induced apoptosis in neurons following spinal cord injury (Zhou et al., 2020), supporting the notion that TFE3 activation could be neuroprotective. Efforts have been made to develop TFEB agonists to enhance autophagy in neurodegenerative disease (Chen et al., 2021), and our results suggest that TFE3 could also be a promising drug target.

Clarification of the mechanism of TFE3 down-regulation in the nucleus might provide a new target for PD neuroprotection as well. Decreased levels of nuclear TFEB, another MiTF/TFE family member, in dopaminergic neurons of PD patients has also been reported (Decressac et al., 2013). MiTF/TFE family members are able to shuttle between the nucleus and the cytoplasm via direct interaction with the 14-3-3 protein (Jin et al., 2004; Bronisz et al., 2006; Martina et al., 2012). mTOR, GSK-3 and CDK4/6 can phosphorylate TFEB and TFE3 to promote their association with 14-3-3 and their retention in the cytosol (Roczniak-Ferguson et al., 2012; Li et al., 2016; Yin et al., 2020). Additionally, increased protein levels of mTOR, GSK-3 and CDK6 have been found in postmortem PD brains (Nagao and Hayashi, 2009; Wills et al., 2012; Mamoor, 2020). Therefore, reduced nuclear TFEB and TFE3 may be due to dysregulation of these kinases in PD. Further studies are needed to reveal the underlying mechanisms, which could provide a new drug target for neuroprotection against PD. Notably, PD is not a single entity (Bloem et al., 2021). Different causes and different mechanisms could be involved in the development of this disease. The findings in this study may not be uniformly applicable to all forms of the disease, or responsible for all of its symptoms.

## Conclusion

In conclusion, our data show for the first time that nuclear TFE3 expression is reduced in the dopaminergic neurons of patients with PD. We also confirm for the first time that TFE3 can regulate the autophagy and survival of dopaminergic neurons, suggesting that reduction of nuclear TFE3 leads to autophagy dysfunction and neuronal death in PD. Furthermore, TFE3 overexpression can activate autophagy to alleviate MPTP-induced neurotoxicity. These findings suggest that TFE3 play a pivotal role in autophagy in PD, and thus it could be a promising drug target for neuroprotective therapy in PD.

## Data Availability

The original contributions presented in the study are included in the article/Supplementary Material, further inquiries can be directed to the corresponding authors.
